# How Are Techno-Stressors Associated with Mental Health and Work Outcomes? A Systematic Review of Occupational Exposure to Information and Communication Technologies within the Technostress Model

**DOI:** 10.3390/ijerph18168673

**Published:** 2021-08-17

**Authors:** Prem Borle, Kathrin Reichel, Fiona Niebuhr, Susanne Voelter-Mahlknecht

**Affiliations:** 1 Charité—Universitätsmedizin Berlin, Corporate Member of Freie Universität Berlin and Humboldt Universität zu Berlin, Institute of Occupational Medicine, Augustenburger Platz 1, 13353 Berlin, Germany; Fiona.Niebuhr@charite.de (F.N.); Susanne.Voelter-Mahlknecht@charite.de (S.V.-M.); 2 Independent Researcher in Occupational Health, 10245 Berlin, Germany; kareichel@gmx.de

**Keywords:** digitalisation, ICT demands, workplace well-being, knowledge workers, platform work, sociotechnical systems, methodology

## Abstract

The technostress model has introduced different factors to consider when assessing how information and communication technologies impact individuals in different work settings. This systematic review gathers evidence regarding associations between occupational exposure to technostress and health or work outcomes. In addition, we highlight typical methodological constraints of the technostress model. We conducted electronic literature searches in June 2020 (PubMed, PubMed Central, Web of Science, Scopus, PsycInfo, PsycArticles) and independently screened 321 articles. We report on 21 articles meeting eligibility criteria (working population, technostress exposure, health or work outcome, quantitative design). The most frequently examined techno-stressors, i.e., factors of technostress, were techno-overload and techno-invasion. Techno-stressors were consistently associated with adverse health and work outcomes, apart from a positive impact on work engagement. However, studies may be subject to considerable conceptual overlap between exposure and outcome measures. Future technostress research would benefit from reducing heterogeneity in technostress measures, assessing their external validity and focussing on specific techno-stressors.

## 1. Introduction

### 1.1. The Technology Paradox

Throughout history, there have been claims that technologies are proliferating and revolutionising work, even if the term digitalisation did not yet exist and the technologies to which it refers keep changing [1]. Public debates typically oscillate between heralding technological good and evil. In practice, however, benefits of technologies may come along with costs [2], which studies in the field of technostress have termed the dark side of technology [3]. In addition to the costs involved in acquiring them [4], there may also be hidden costs for worker satisfaction and health. These are contextually influenced and therefore a scientific challenge to classify, quantify and thus make visible [4].

Foundational empirical studies with the technostress model found clear associations with lower performance [5], lower job satisfaction [6] and higher strain [7]. Despite a recent attempt to introduce notions of positive implications of information and communication technologies (ICT) [8], technostress research has mostly focused on the negative consequences resulting from work-related ICT use [9]. In contrast, field studies of human–machine interaction from a sociotechnical perspective have sought to uncover how the effects of technologies are ambiguous, potentially paradoxical. Mobile device use for work allows remote workers to use mobile devices to perform work *anywhere/anytime*, which is why remote work is commonly framed and experienced as increasing personal autonomy. However, workers may begin to feel like they are forced to use it *everywhere/all the time*, thereby simultaneously diminishing their sense of autonomy. Mazmanian et al. call this “the autonomy paradox” [10]. 

### 1.2. The Technostress Model

The technostress model has gained immense popularity and established itself in high-impact fields of computer science, management and psychology [11]. Studies on technostress have focused on a set of factors measuring potentially negative implications of technology use. Five so-called techno-stressors were introduced and validated within the framework of the technostress model [5,6]: techno-overload (technology forces workers to work more and faster); techno-invasion (invasion of private life due to technology that creates pressures of constant connectivity); techno-complexity (technology is complex, leading to a sense of lack with regard to computer skills); techno-insecurity (workers feel threatened about losing their jobs because of new technologies); techno-uncertainty (constant technological changes that may create stress for workers). A modified technostress model was proposed, but has been less frequently replicated [7]. Proving to be highly flexible and widely applicable, a range of studies apply techno-stressors to operationalise both exposures or outcomes, enabling the core subconstructs to rapidly become scientific convention and popular across disciplinary borders [12]. 

In this systematic review, we narrow the focus down to studies that consider these techno-stressors as exposures that have effects on work and health outcomes. In contrast to previous reviews, this focus enables greater comparability between studies and of the synthesised findings. The data collection and analyses extend and build on the research gap and recommendation with which the recent review by LaTorre et al. (2019) [12] concludes: “studies should focus on a single aspect of technostress, rather than simultaneously approaching multiple stressors and consequences.” Given that review studies to date—as well as most original studies—rarely distinguish measures of overall technostress and specific techno-stressors when reporting studies and their methods and findings [12], there is a lack of collated data for researchers to assess the promises and perils of specific techno-stressors.

Despite noting a lack of detailed reporting of measurement methods, Fischer and Riedl [13] suggested a characterisation of technostress research as a “nurturing ground for measurement pluralism”. However, as “measurement pluralism” may also limit the potential for further research to distinguish the implications of specific techno-stressors, it needs to be acknowledged as a methodological gap that leads to conceptual blind spots. In what follows, this systematic review aims to close this gap by answering the following research questions: First, how is work-related exposure to specified techno-stressors associated with health or work outcomes? Second, do methodological issues in applications of the technostress mode, to date, limit its conceptual scope regarding specific techno-stressors?

## 2. Materials and Methods 

### 2.1. Search Strategy and Study Selection

This systematic review follows the PRISMA statement guidelines [14] and we registered a review protocol in the PROSPERO database (CRD42020199960). More detailed information on the search strategy can be found in our previous publication based on our research process [15]. This systematic review is the second paper to result from one review and data collection process. The first paper [14] used identical search strings and databases (Appendix B). However, the underlying research questions differ significantly. While the present paper investigates the extent to which work-related exposure to specific techno-stressors is associated with health and work-related outcomes, in the previous paper, we examined the generalisability of study results on technostress with a focus on socioeconomic factors [14]. We focused on the extent to which samples in studies of technostress represented variation with respect to sociodemographic characteristics. Thus, both papers are based on the same review process, but they differ in their focus, data analyses, and results.

The systematic literature searches took place in June 2020 and covered the interdisciplinary databases PubMed, Pub Med Central, Web of Science, Scopus, PsycInfo, and PsycArticles. Mattioli and colleagues [16] used a basic search string for research related to occupational health. We used this validated search string to specify our systematic search to the work environment. To identify missing relevant publications, we manually searched in reference lists of review articles. A total of 321 articles were independently reviewed based on title and abstract by two reviewers (PB and KR). Of these, 50 articles were included in the full-text screening, which was performed independently by both reviewers using Rayyan [17]. In case of disagreement with study selection between the two reviewers, a third reviewer (SVM) was consulted.

We defined eligibility criteria based on the PICOS scheme. The study population had to consist of working adults; exclusion took place for samples not related to work and for study populations of employees under 18 years. For the included studies, the study populations were required to be working adults exposed to technostress related to ICT use for work purposes. In studies that measured constructs differently from items in the technostress model, we assigned the techno-stressor that corresponds most. This is considered analytically distinct from non-work ICT use [12,18]. Regarding outcomes, studies were included that examined mental health and personal work outcomes (e.g., job satisfaction and/or job burnout), as both are often studied in relation to technostress [19]. Finally, quantitative studies with cross-sectional or longitudinal design reporting original data were selected. Consequently, non-empirical studies (e.g., commentaries, policy briefs) and qualitative or experimental studies as well as publications with unclear study design were excluded. The eligible publications had to be peer reviewed and available in English. 

### 2.2. Data Collection and Quality Appraisal 

Both reviewers (PB und KR) defined the key topics for data extraction and one reviewer (PB) afterwards extracted the data. In the case of studies without information on the response rate, we contacted the authors. We used the Newcastle–Ottawa scale (NOS) adapted for cross-sectional studies (Herzog et al., 2013) to assess the risk of bias in the included studies. The NOS has been previously applied in systematic reviews of technostress [12,20]. According to this instrument, studies are rated on a scale of 0–10 stars on the dimensions selection, comparability, and outcome. Here, high scores represent low risk of bias: high risk (score 0–3), medium risk (score 4–6), and low risk (score 7–10). 

## 3. Results

**Figure 1:**
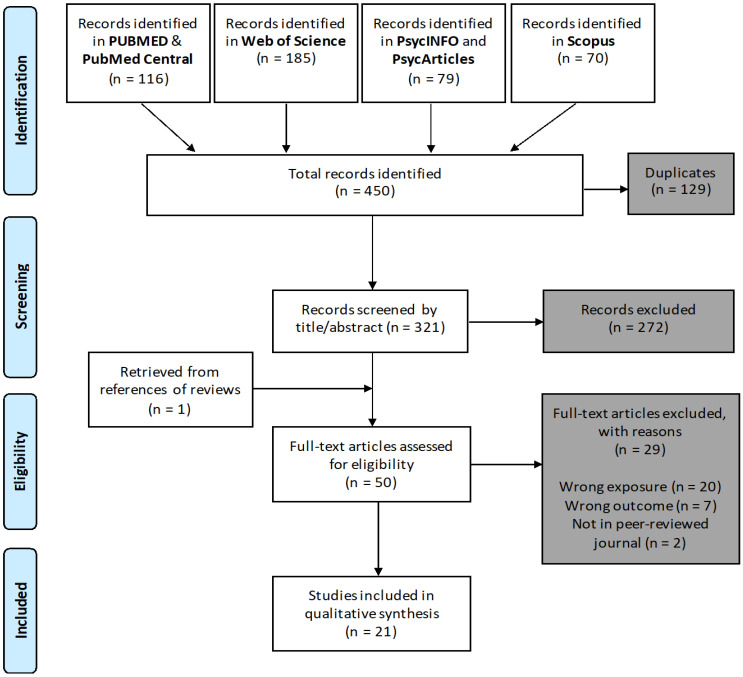
Preferred Reporting Items for Systematic Reviews and Meta-Analyses (PRISMA) flow diagram depicting the number of reports screened and included in this systematic review. Source: [15].

The process of study selection is shown as a PRISMA flow diagram in Figure 1. In total, our searches of four multidisciplinary databases identified 450 records. After de-duplication according to validated steps [21], 321 articles were screened based on title and abstract. An additional article was identified from references of the review articles. Full-text screening was performed on 50 articles.

During the full-text screening, a total of 20 articles were excluded because they measured a different exposure. In addition, seven articles were excluded because of measuring a different outcome, and two because they were published in journals without peer review. A complete summary of the 21 articles included in our systematic review is provided as supporting information (Appendix A). The included studies contained considerable heterogeneity in the selection and analysis of ICT exposure and outcome measures. We found that when operationalising ICT exposures with the technostress model, techno-overload was the most commonly examined techno-stressor in 17 studies (81% of studies), followed by techno-invasion in 16 studies (76%), techno-insecurity in 11 studies (52%), techno-complexity in 8 studies (38%) and techno-uncertainty in 7 studies (33%), as previously described elsewhere [15]. The exposure measures, sampled occupations, and reported outcome measures varied substantially. Therefore, this paper focuses on describing and assessing the studies, their results, their applicability, and their limitations through a qualitative synthesis in the following sections: quality appraisal sections (Section 3.1) study characteristics sections (Section 3.2), health outcomes sections (Section 3.3) and work outcomes sections (Section 3.4).

### 3.1. Quality Appraisal 

Using the Newcastle–Ottawa scale (NOS) adapted for cross-sectional studies by Herzog et al. [22], we assessed the methodological quality of the included studies, whereas higher scores indicate better quality. All studies used self-report questionnaires to examine exposure to technostress and other outcomes. Scores given ranged from 3 to 9 (*Mdn* = 6), with two studies demonstrating high risk of bias, ten studies a medium risk, and nine studies low risk (see Appendix A). The highest score (NOS = 9) was achieved by two studies based on the same representative dataset from Sweden [23,24]. The studies by Ayyagari et al. [7] and Goetz and Boehm [25] were based on simple randomised samples and received a score of 8. 

Of the 21 included studies, 20 were cross-sectional and 16 were based on convenience samples. Regarding response rates, three of the five randomised study samples reported satisfactory response rates, whereas studies using commercial online panels did not report response rates. The majority of studies considered at least one potential confounder, while seven studies did not describe any confounders. Furthermore, an additional analysis regarding potential sociodemographic differences in included study populations was carried out and reported separately with a focus on limitations to the generalisability of study findings and the role of socioeconomic position [15].

### 3.2. Study Characteristics

As shown in Appendix A, a majority of six studies were conducted in the USA and fourteen were published in journals for Information Systems, i.e., ICT-oriented business research [15]. The 16 convenience samples were collected in diverse organisations with a range from 152 to 14,757 employees. The five randomised samples varied in the number of employees from 374 to 14,757. Reported response rates ranged from 16 to 89%. The age distribution and gender ratio were not balanced in most studies, and the latter varied depending on the sample settings. The overrepresentation of age or gender groups varied enormously between most studies. 

Following the technostress model (i.e., a distinction of techno-stressors), the different terminology and measurement items for technostress exposure were considered. On the one hand, only six of the 21 studies measured exposure to all subconstructs of technostress. On the other hand, six studies focused on just one techno-stressor. We classified the examined outcomes into health (Table 1) and work outcomes (Table 2). Nine studies analysed exclusively health outcomes, seven studies examined exclusively work outcomes and five studies examined both types of outcomes.

### 3.3. Associations with Health Outcomes

Of the 21 included studies, 14 examined at least one of five different health outcomes, i.e., self-rated health (3), strain and stress (4), negative emotion and anxiety (3), burnout (2) and work exhaustion (2). Of the three studies analysing *self-rated health*, all achieved high NOS scores (from 8 to 9, i.e., good quality) and two were based on nationally representative samples in Sweden. Stadin, Nordin, Broström, Magnusson Hanson, Westerlund and Fransson [24] found negative associations between techno-overload, operationalised as ICT demands, and self-rated health. A later follow-up study used longitudinal analyses to show a long-term reduction in self-rated health, which persisted after adjustments for age, sex, socioeconomic position (SEP), health behaviours, body mass index, job strain and social support [23]. The third study, a time-lagged analysis of a simple randomised sample from a commercial online panel, found a significant direct and negative effect of techno-insecurity on self-rated health measured at a later point in time [25]. 

Four studies examined the health outcomes *strain* or *stress*. A study with a high NOS score based on a simple randomised sample from a commercial online panel [7] showed that techno-overload, techno-invasion and techno-insecurity were positively associated with strain. A study of teleworkers with a medium NOS score by [26] reported that techno-overload and techno-invasion led to greater strain, which in turn reduced teleworkers’ job satisfaction. Another study with a medium NOS score in a sample of workers using human resource technologies, Florkowski [27], found techno-insecurity was positively associated with work stress. Day et al. [28] scored high on the NOS and operationalised technostress as ICT demands, which includes a subconstruct similar to techno-overload and reported associations with ICT stress and strain. Their study shows that techno-overload was most strongly associated with increased ICT stress, also after controlling for demographics, job variables, and job demands. In their sample consisting of multiple professions, techno-overload was not associated with strain. 

Three studies examined the health outcomes related to *negative emotions*. Techno-overload [29], techno-invasion [30] and both techno-overload and techno-invasion combined [31] were shown to be associated with increased negative emotions (e.g., measured as anger, anxiety and nervousness). However, all three studies scored low to medium quality on the NOS.

Two studies examined the health outcome *burnout*. In their international study of senior managers and executives that achieved a high NOS score, Srivastava, Chandra and Shirish [8] found all techno-stressors together were positively associated with job burnout. In their study with a medium NOS score, Khedhaouria and Cucchi [32] identified multiple configurations that led to positive associations between techno-overload, techno-invasion, techno-insecurity and burnout. Individuals reported high burnout levels when technostress situations were characterised by the following configuration: low invasion of privacy, high role ambiguity, and high job insecurity; low invasion of privacy, high work overload, role ambiguity, and job insecurity; high invasion of privacy, work overload, and role ambiguity; and high work overload and role ambiguity.

Finally, two studies examined the health outcome *work exhaustion*. In their study with a medium NOS score, Kim et al. [33] found that techno-overload, techno-invasion and techno-insecurity combined were positively associated with work exhaustion in mobile workers. However, when analysing techno-stressors individually, the effects of overload and insecurity were no longer significant. The study by Gaudioso et al. [34], which scored high on the NOS, reported that techno-overload and techno-invasion were positively associated with work exhaustion. This study also showed that the effects were mediated by strain facets and coping strategies. 

**Table 1:** Findings on health outcomes (sorted by NOS score).

First Author and Year	Techno-Stressor(s)	Health Outcome ^a^	Findings Health ^a^	*N*	Confounders	NOS Score ^b^
Stadin et al. [23] 2019	Techno-Overload Techno-Invasion (operationalised as ICT demands)	self-rated health (−)	Repeated exposure to overload was associated with increased risk of suboptimal SRH in the crude analysis (OR 1.36, CI [1.11–1.67]), also after adjustments (OR 1.34, CI [1.06–1.70]). Repeated exposure to high techno-overload was most prevalent among participants with high (40%), followed by participants with intermediate (35.5%) and low SEP (12.5%).	4.468	sex, age, SEP, health behaviours, BMI, job strain, social support	9 ^c^
Stadin et al. [24] 2016	Techno-Overload Techno-Invasion (operationalised as ICT demands)	self-rated health (−)	Overload and invasion were associated with suboptimal self-rated health in crude analysis (OR 1.35 [CI 1.24–1.46]) also after adjustments (OR 1.49 [CI 1.36–1.63]). ICT demands were most prevalent among participants with high SES (59.8%), followed by participants with intermediate SES (54.9%) and low SES (29.1%).	14.873	sex, age, SEP, lifestyle factors	9
Goetz and Boehm [25] 2020	Techno-Insecurity	self-rated health (−)	There was a significant time-lagged negative effect of insecurity on general health (B = −0.27, *p* < 0.001; *F* = 81.47, *df* = 3, *p* < 0.01). *F* = 11.84 **; *R*² (Fishers-z) = 0.125.	8.019	educational level, job position, age, gender disability status, working hours per week, organisational tenure, physical activity, occupational segment, companies’ operating sector	8
Ayyagari et al. [7] 2011	Techno-Overload, Techno-Insecurity, Techno-Invasion	strain (+)	Overload (β = 0.26, *p* < 0.01) and insecurity (β = 0.10, *p* < 0.01) are positively associated with strain. Invasion is not associated with strain (β = 0.027, *p* > 0.05).	661	negative affectivity, technology usage	8
Day et al. [28] 2012	Techno-Overload (operationalised as ICT demands)	strain, (n.s.) stress (+)	Overload was associated with increased stress (β = 0.26, *p* < 0.01). Overload was not associated with strain (β = 0.06, *p* < 0.01). ICT support was associated with better health outcomes.	258	age, gender, job tenure, of technologies used	7
Gaudioso et al. [34] 2017	Techno-Overload, Techno-Invasion	work exhaustion (+)	Overload (*r* = 0.50 **) and invasion (*r* = 0.35 **) were associated with increased work exhaustion. Significant indirect effects on work exhaustion of techno-overload (*CI*_95_ [0.154; −0.338], *p* < 0.004) and techno-invasion (*CI*_95_ [0.128; −0.356], *p* < 0.012). The effects are mediated via strain facets and coping strategies.	242	age, gender, daily hours of work	7
Srivastava et al. [8] 2015	All	burnout (+)	Techno-stressors are positively associated with job burnout (β = 0.324, *p* < 0.01).	152	age, gender, location, experience, job demand, job control	7
Khedhaouria and Cucchi [32] 2019	Techno-Overload, Techno-Invasion, Techno-Insecurity	burnout (+)	Individuals may experience high job burnout when technostress situations are characterised by low invasion of privacy, high role ambiguity, and high job insecurity; low invasion of privacy, high work overload, role ambiguity, and job insecurity; high invasion of privacy, work overload, and role ambiguity; and high work overload and role ambiguity.	161	n. a.	6
Kim et al. [33] 2015	Techno-Overload, Techno-Invasion, Techno-Insecurity, Techno-Complexity	work exhaustion (+)	Overall techno-stressors (β = 0.327 ***) are positively associated with work exhaustion in mobile enterprise environments and work exhaustion is negatively associated with job satisfaction. Overload (β = 0.119, n.s.); invasion (β = 0.224 **); Insecurity (β = −0.019, n.s.); complexity (β = 0.107 ***).	210	age, gender, duration, job stressors	6
Wu et al. [30] 2020	Techno-Invasion	negative emotion (+)	Invasion can significantly predict anxiety, (β = 0.27, *p* < 0.01).	374	age, gender, work experience, education, income, position	6
Suh and Lee [26] 2017	Techno-Overload, Techno-Invasion	strain (+)	Overload (β = 0.23 **) and invasion (β = 0.11 **) are positively associated with strain, which in turn reduces teleworkers’ job satisfaction (β = 0.40 ***)	258	age, gender, education	5
Florkowski [27] 2019	Techno-Insecurity	stress (+)	Insecurity is positively associated with work stress (β = 0.37 ***)	676	no analyses of confounders despite collecting sociodemographic data	4
Lee [35] 2016	Techno-Overload	negative emotion (+)	Overload is positively associated with negative emotion, i.e., anger (β = 0.622 **) and anxiety (β = 0.661 **)	222	age, gender	4
Jena [31] 2015	Techno-Overload, Techno-Invasion	negative emotion (+)	Overload and invasion are negatively associated with negative emotion (β = 0.25 **)	216	none	3

^a^ Negative and positive associations are represented by −/+, respectively. * *p* < 0.05, ** *p* < 0.01, *** *p* < 0.001. n.s. indicates results were not significant. We report R^2^ only if the variance relates to techno-stressors. ^b^ NOS score out of 10 possible points. **^c^** As the scoring scale would differ and reduce comparability of scores, we report the adapted NOS, noting the longitudinal design of the study would score 2 additional points: 1 for measuring the outcome at the start of study and 1 for a follow-up of >1 year.

### 3.4. Associations with Work Outcomes

As Table 2 shows, twelve studies examined at least one of three different work outcomes, i.e., job satisfaction (6), productivity or performance (5) and work engagement (2). Of the six studies analysing *job satisfaction*, the foundational study by Ragu-Nathan, Tarafdar, Ragu-Nathan and Tu [6], which scored 6 with regard to NOS, established that overall technostress was negatively associated with job satisfaction. A later study showed that all the individual subconstructs were negatively associated with job satisfaction [36]. The latter study also found that respondents generally reported low levels of techno-insecurity, but only achieved a NOS score of 3.

Multiple studies focusing on either techno-overload, techno-invasion, techno-insecurity and techno-complexity [33], techno-overload and techno-invasion among teleworkers [26], techno-overload and techno-invasion [31] or techno-insecurity [27], all found that the respective techno-stressors were associated with reduced job satisfaction. It should also be noted, however, that these studies achieved medium or low NOS scores.

Five studies examined associations with *productivity* or *performance*, achieving relatively low NOS scores between 3 and 5. Three found that all techno-stressors were negatively associated with productivity [5,37,38]. A study focusing on techno-overload and techno-invasion found them to be negatively associated with performance [31] and another study found techno-overload, techno-complexity and techno-uncertainty to be negatively associated with productivity in the aviation industry [39].

Two studies examining associations with *work engagement* in a sample of executives and managers achieved a comparatively good NOS score of 7. However, they examined different technostress exposures, which resulted in differing implications of technostress. When measuring the overall exposure to all techno-stressors, technostress was shown to be positively associated with work engagement (i.e., higher technostress was associated with increased work engagement) [8]. In addition to the multitude of negative effects of techno-stress described here, it is noteworthy to highlight the positive effect found for techno-stress creators on work engagement. The authors explain the effects found with the idea that employees might see techno-stress creators as an opportunity or challenge and that this might thus increase their engagement at work [8]. When measuring only techno-invasion and grouping respondents into different levels of technology use, managers with an intensive use reported negative associations with work engagement [40]. A comparison of two groups of different occupational positions (senior executives vs. middle managers), revealed no significant differences between both groups [40].

**Table 2:** Findings on work outcomes (sorted by NOS score).

First Author and Year	Techno-Stressor(s)	Work Outcome ^a^	Findings Work	Covariates	NOS Score ^b^
Vayre and Vonthron [40] 2019	Techno-Invasion	work engagement (−)	Invasion is negatively associated with work engagement among executives and managers with high intensive use.	age, personal uses differences, socio-professional characteristics	7
Srivastava et al. [8] 2015	All	work engagement (+)	Techno-stressors are positively associated with work engagement among executives and managers (β = 0.186, *p* < 0.05).	age, gender, location, experience, job demand, job control	7
Ragu-Nathan et al. [6] 2008	All	job satisfaction (−)	Techno-stressors are negatively associated with job satisfaction (β = −0.13, *p* <0.01).	none	6
Kim et al. [33] 2015	Techno-Overload, Techno-Invasion, Techno-Insecurity, Techno-Complexity	job satisfaction (−)	Techno-stressors are positively associated with work exhaustion (β = 0.327, *p* < 0.01) in mobile enterprise environments, which in turn reduces job satisfaction (β = −0.149, *p* < 0.05).	age, gender, duration, job stressors	6
Suh and Lee [26] 2017	Techno-Overload, Techno-Invasion	job satisfaction (−)	Overload and invasion among teleworkers are negatively associated with strain, which in turn reduces job satisfaction (β = −0.040 *p* < 0.01). The manner in which technology and job characteristics influence teleworkers’ technostress varies depending on the intensity of teleworking.	age, gender, and education	5
Alam [39] 2016	Techno-Overload, Techno-Complexity, Techno-Uncertainty	productivity (−)	Overload (β = −0.311, *p* < 0.05), complexity (β = −0.348, *p* < 0.05), and uncertainty (β = −0.165, *p* < 0.1), are negatively associated with productivity in aviation (*R*^2^ = 0.49 *).	none	5
Tarafdar et al. [37] 2015	All	productivity (−)	Techno-stressors are negatively associated with productivity among salespersons (β = −0.147, *p* < 0.05).	education, organisational tenure, professional tenure	5
Tarafdar et al. [5] 2007	All	productivity (−)	Techno-stressors are negatively associated with productivity (β = −0.280, *p* < 0.01).	none	4
Tarafdar et al. [38] 2011	All	productivity (−)	Techno-stressors are negatively associated with productivity (β = −0.33, *p* < 0.01).	organisation	4
Florkowski [27] 2019	Techno-Insecurity	job satisfaction (−)	Techno-insecurity is negatively associated with job satisfaction (β = −0.27, *p* < 0.01).	none	4
Al-Ansari and Alshare [36] 2019	All	job satisfaction (−)	Techno-stressors decrease employee’s job satisfaction (β = −0.25, *p* < 0.01, *R*^2^ = 0.27). All sub-constructs were significant. Respondents reported low insecurity, i.e., fear of being replaced or unemployed by new technologies or by other employees who have a better understanding of new technologies.	none	3
Jena [31] 2015	Techno-Overload, Techno-Invasion	job satisfaction (−) performance (−)	Overload and invasion are negatively associated with job satisfaction (β = −0.41, *p* < 0.01) and performance (β = −0.33, *p* < 0.05).	none	3

^a^ Negative and positive associations are represented by -/+, respectively. * *p *< 0.05, ** *p* < 0.01, *** *p* < 0.001. n.s. indicates not significant results; ^b^ NOS score out of 10 possible points.

## 4. Discussion

### 4.1. Health and Work Outcomes

Overall, the included studies comprised a variety of techno-stressors and their associations with health and work outcomes in the context of the technostress model. In comparison, health and work outcomes appear equally applicable to develop a broader understanding of the ramifications of technostress, although the NOS scores were slightly better among the studies with work outcomes. However, only one study with health outcomes compared to six studies with work outcomes measured the exposure to all subconstructs of technostress. When more than one techno-stressor is measured, studies mostly analyse and report composite scores of technostress. This pattern in reporting findings on technostress limit the potential of assessing the role of individual techno-stressors [12].

All techno-stressors combined were associated with increased burnout. Individual techno-stressors also consistently showed adverse implications with regard to self-rated health, strain or stress, negative emotions, burnout and work exhaustion. [33] In one study [33], techno-overload, techno-invasion, and techno-insecurity were only significantly associated with exhaustion when analysed as a composite score. When analysing techno-stressors individually, the effects of overload and insecurity were no longer significant, which demonstrates the importance of assessing the role of individual techno-stressors. Although negative emotions displayed greater risk of bias than other outcomes examined in our review, studies that examined technostress as an outcome measure have also reported negative implications regarding negative emotion [3]. 

A better understanding of work outcomes of technostress and constructs associated with technology is important to developing effective interventions. Among the included studies examining associations of technostress with work outcomes, all but one study demonstrated that increased technostress is associated with adverse work outcomes [8]. Technostress consistently had an adverse impact on job satisfaction and productivity/performance, while work engagement was both negatively and positively associated in different studies. Molino et al. [41] found in their study focusing on blue-collar workers that technology acceptance was positively associated with work engagement. Thus, the level of technology acceptance in a sample of workers may play a role in explaining such different findings between studies.

Srivastava, Chandra and Shirish [8] claim to be one of the first to empirically show that increased technostress was associated with an improved work outcome. When moderated by the Big Five personality trait openness, increased technostress was associated with increased work engagement. Both studies on work engagement are comparable in terms of average age and the samples of senior managers and executives, who represent highly qualified workers [8,40]. However, the terminology and measurement of technostress exposure and its outcomes has not been consistent across the studies. Potentially relevant differences could be explained with a significantly larger proportion of male respondents (76%, see Appendix A) in the study of combined techno-stressors [8], compared to an almost equal gender distribution in a study reporting the adverse association of techno-invasion on work engagement [40]. Nonetheless, the findings regarding work engagement suggest that some associations may be applicable specifically to highly qualified workers. 

The potential for positive effects of technostress has recently been emphasised in a study that aimed to introduce the concept of techno-eustress based on stress research [9]. Similarly, Vayre and Vonthron [40] decidedly applied a differentiated approach that does not a priori assume techno-invasion to be negative. An older study of telework found stress to be improved and suggested it may already be accepted in advance that work could interfere with family life [42]. The study did not find gender differences and was solely based on a sample of managers and sales professionals representing highly qualified workers.

Overall, several of the discussed studies were based largely on convenience samples of highly qualified workers, thus the potentially positive implications of telework and techno-invasion may be influenced by socioeconomic factors. Similarly, some potentially positive implications of technostress for work engagement may also be influenced by socioeconomic factors, although the findings have been inconclusive. Potential sociodemographic differences regarding specific techno-stressors have been suggested elsewhere [15].

### 4.2. Conceptual Developments

Recent theoretically-inspired interventions have advocated a sociotechnical framework [2,43]. Important scholars in the field of technostress have picked up this emphasis on conceptual development by drawing on social theory [9,37]. Nonetheless, the technostress model has not yet achieved the same level of popularity in the social sciences as it has in the field of Information Systems. Building on the call in Ayyagari, Grover and Purvis [7] to develop a taxonomy or typology for ICT, we would recommend aiming for a model of ICT exposure at work that captures situated interactions over time and can guide research decisions as to which techno-stressors are relevant in a given work setting. In a similar vein, multiple studies have proposed conceptual developments that aim to capture moderating effects of psychological characteristics like self-efficacy [44], time management [45] and coping strategies [46], which may have a positive influence with regard to technostress. These studies delineate potential mechanisms to prevent or reduce technostress. Moreover, work design may provide a conceptual framework to capture and assess contextual factors that can result in both positive and negative implications of technostress [47].

More context-specific conceptual development would redress the one-dimensional negative wording in the technostress model that underlies the proclivity for conceptual overlap and inflate associations. For instance, studies should aim to distinguish techno-invasion as a negative implication and increased autonomy as a positive implication of remote or mobile work [10]. It may also be useful to develop concepts that aim to look at interconnections between broader social and technological trends [48]. The field could draw more on public health and social sciences, which may help to better distinguish paradoxical implications of technologies.

### 4.3. Limitations

Our findings are subject to the following limitations. First, although the studies included in our review found consistent statistically significant associations between technostress and the outcomes of interest, the summary conclusions could have been influenced by publication bias, i.e., the non-publication of non-statistically significant findings [49,50]. Moreover, as most of the included studies did not follow a longitudinal design, the relations found cannot be interpreted as causal effects. 

Second, many studies may not use the term “technostress” when researching ICT use at work and may therefore not have been identified in our search strategy. Third, this review focused on technostress as an exposure variable. Other studies that employed technostress as an outcome variable have not been examined in this review, although they are also relevant to better understand the role of specific techno-stressors. Our review also suggests that technostress is hard to differentiate as either an exposure or outcome variable and that conceptual issues regarding exposure and outcome variables need to be addressed.

Fourth, both measures of technostress exposure and outcomes were based on self-reported measures. Moreover, the very high reliability of associations within the technostress model may indicate issues due to conceptual overlap between survey items measuring technostress or ICT demands [23,24] and health outcome measures. In other words, a negative or positive perception of one’s health or work may influence survey responses to both exposure and outcome measures. To the extent that this is the case, it raises potential limitations regarding the external validity and generalisability of the technostress model. Future systematic reviews could expand their scope to cover biological or physiological health measures [51,52]. 

Finally, as previously mentioned, this review is based on an analytical approach very similar to our previous review study [15]. As each literature review process has the potential for bias, multiple uses of closely related review data may thus entail risks for the synthesis of evidence and drawing of conclusions. Overall, the conclusions in this study and our previous publication are based on systematic literature searches until June 2020 and should be considered together.

## 5. Conclusions

First, this systematic review showed consistent negative implications of all techno-stressors with health and work outcomes, except for one positive association with work engagement. Thus, work engagement may be positively influenced by techno-stressors. However, the study did not report associations by individual techno-stressors. Future research on technostress would benefit from analysing and reporting associations on the level of specific techno-stressors. To date, techno-overload and techno-invasion have received the most attention. Furthermore, as techno-stressors can quickly become outdated, repeated conceptual work is required to include new phenomena. To assess the applicability of old and new factors of technostress, the individual associations of all subconstructs need to be reported.

Second, this review shows that although studies refer to technostress in general, upon closer examination, they often measure an insufficiently specified subset of techno-stressors. Consequently, many studies of technostress risk implicitly generalising their findings to overall technostress, even if they only examined exposures to individual subconstructs of technostress. A question that remains to be elaborated is whether there are inhibiting factors such as methodological issues (e.g., null findings) that result in the preference for analysing only a composite score of technostress. Such conceptual conflation may simultaneously facilitate and constrain scientific and interdisciplinary exchange. 

Based on theoretical and practical considerations, future studies should consider whether in a specific study sample and context it makes more sense to later analyse technostress as a composite score or rather an individual techno-stressor. This aspect is relevant for practitioners as it means that measurement tools should capture both negative and positive outcomes of technological changes. To this end, tools have to be sufficiently context specific rather than just aiming for a general universal measure. 

A challenge for quantitative study designs is to methodologically take into account that there could be positive implications of specific techno-stressors depending on ambiguous factors [40]. The implications of technostress may differ when measuring constructs such as work–life conflict [40] or work–life balance [44] instead of techno-invasion or work intensification [48] instead of techno-overload. There is vast research on potentially more context-sensitive constructs similar to individual techno-stressors (e.g., work intensification, work–life conflict), but that do not fully match the either routinely applied or selected survey items from the technostress model. These methodological decisions should be reported in sufficient detail to trace the conceptual changes they represent, not least to ensure comparability and reproducibility. 

Consequently, to generate a better knowledge base regarding the implications of specific techno-stressors, technostress research should examine relations to similar research fields with different, but related, constructs. Although the popular technostress model has been repeatedly validated, researchers may need to recognise the value of potentially less validated constructs. This would require greater interdisciplinary exchange, which would be part and parcel of establishing the external validity of the technostress model and its subconstructs. Not every techno-stressor may be relevant in each work setting, which means that studies should aim to pre-assess the relevance of specific techno-stressors rather than trying to uncritically replicate the technostress model. Last but not least, study designs with technostress should be able to account for the findings from paradoxical effects of technologies.

## Data Availability

No new data were created or analysed in this study. Data sharing is not applicable to this article.

## Appendix A

**Table A1:** List of included studies.

First Author and Year	Country Data	Journal Discipline	Sample Type	N (RR) ^a^	Gender (% men ^b^)	Age	NOS Score	Techno- Overload (17)	Techno-Invasion (16)	Techno-Insecurity (11)	Techno-Complexity (8)	Techno-Uncertainty (7)
Al-Ansari and Alshare [36]	Qatar	Information Systems	convenience	410 (−)	33%	Below 31 (37%); between 31 and 40 (32%); over 41 (31%)	3	x	x	x	x	x
Alam [39]	Pakistan	Information Systems	randomised	203 (68%)	82%	Not reported	5	x			x	x
Ayyagari, Grover and Purvis [7]	USA	Information Systems	randomised (online panel)	661 (−)	52%	*M* = 49 *Mdn* = 52	8	x	x	x		
Day, Paquet, Scott and Hambley [28]	Canada	Psychology	convenience	258 (−)	47%	*M* = 35	7	x				
Florkowski [27]	USA	Information Systems	convenience	177 (22%)	not reported	Not reported	4			x		
Gaudioso, Turel and Galimberti [34]	USA	Psychology	convenience	242 (16%)	28%	Modal age bracket: 45–54 years	7	x	x			
Goetz and Boehm [25]	Germany	Information Systems	randomised (online panel)	8019 (59%)	51%	*M* = 44.3 (range: 18–77)	8			x		
Jena [31]	India	Information Systems	convenience	216 (54%)	54%	over 35 (53%); below 35 (47%)	3	x	x			
Khedhaouria and Cucchi [32]	France	Information Systems	convenience	465 (46%)	50%	*M* = 39 (range: 24–62)	6	x	x	x		
Kim, Lee, Yun and Im [33]	South Korea	social sciences	convenience	210 (−)	70%	over 40 (60%)	6	x	x	x	x	
Lee [29]	South Korea	Psychology	convenience	222 (−)	81%	20–29 (1.7%), 30–34 (17.6%), 35–39 (17.1%), 40–44 (35.1%), 45–59 (22.5%)	4	x				
Ragu-Nathan, Tarafdar, Ragu-Nathan and Tu [6]	USA, India	Information Systems	convenience	608 (89%)	63%	below 45 (>50%)	8	x	x	x	x	x
Srivastava, Chandra and Shirish [8]	International	Information Systems	convenience	152 (22%)	76%	*M* = 37.96 (*SD* = 6.73)	7	x	x	x	x	x
Stadin, Nordin, Broström, Magnusson Hanson, Westerlund and Fransson [24]	Sweden	Public health	randomised (nationally representative)	14,757 (53%)	43%	Range: 20–75, younger people underrepresented	9	x	x			
Stadin, Nordin, Brostrom, Hanson, Westerlund and Fransson [23]	Sweden	Public health	randomised (nationally representative)	4468 (61%)	43%	*M* 47.3 Most 40–59	9	x	x			
Suh and Lee [26]	South Korea	Information Systems	convenience	258 (86%)	57%	Not reported	5	x	x			
Tarafdar, Tu, Ragu-Nathan and Ragu-Nathan [5]	USA	Information Systems	convenience	237 (47%)	17%	Not reported	4	x	x	x	x	x
Tarafdar, Tu and Ragu-Nathan [38]	USA	Information Systems	convenience	233 (73%)	17%	Not reported	4	x	x	x	x	x
Tarafdar, Pullins and Ragu-Nathan [37]	UK	Information Systems	convenience	233 (73%)	66%	Range: 26–56	5	x	x	x	x	x
Vayre and Vonthron [39]	France	Psychology	convenience	502 (67%)	ca. 50%	*M* = 40	7		x			
Wu, Wang, Mei and Liu [30]	China	Information Systems	randomised (online panel)	374 (62%)	42%	26–35 (75%)	6		x			

^a^ Response rate. ^b^ Men are reported as reference because several studies only reported the proportion of men in the sample without missing values.

## Appendix B

**Table A2:** Search strategy (PubMed and PubMed Central, slightly adapted for other databases).

(i) (occupational diseases [MH] OR occupational exposure [MH] OR occupational exposure * [TW] OR “occupational health” OR “occupational medicine” OR work-related OR working environment [TW] OR at work [TW] OR work environment [TW] OR occupations [MH] OR work [MH] OR workplace * [TW] OR workload OR occupation * OR worke * OR work place * [TW] OR work site * [TW] OR job * [TW] OR occupational groups [MH] OR employment OR worksite * OR industry)	AND	(ii) (technostress OR techno-stress)]

## References

[1] Wajcman J. (2015). Pressed for Time: The Acceleration of Life in Digital Capitalism.

[2] Orlikowski W.J., Iacono C.S. (2001). Research commentary: Desperately seeking the “IT” in IT research—A call to theorizing the IT artifact. Inf. Syst. Res..

[3] Salanova M., Llorens S., Cifre E. (2013). The dark side of technologies: Technostress among users of information and communication technologies. Int. J. Psychol..

[4] Star S.L., Strauss A. (1999). Layers of Silence, Arenas of Voice: The Ecology of Visible and Invisible Work. Comput. Supported Coop. Work CSCW.

[5] Tarafdar M., Tu Q., Ragu-Nathan B.S., Ragu-Nathan T.S. (2007). The impact of technostress on role stress and productivity. J. Manag. Inf. Syst..

[6] Ragu-Nathan T.S., Tarafdar M., Ragu-Nathan B.S., Tu Q. (2008). The Consequences of Technostress for End Users in Organizations: Conceptual Development and Empirical Validation. Inf. Syst. Res..

[7] Ayyagari R., Grover V., Purvis R. (2011). Technostress: Technological antecedents and implications. MIS Q. Manag. Inf. Syst..

[8] Srivastava S.C., Chandra S., Shirish A. (2015). Technostress creators and job outcomes: Theorising the moderating influence of personality traits. Inf. Syst. J..

[9] Tarafdar M., Cooper C.L., Stich J.-F. (2019). The technostress trifecta—techno eustress, techno distress and design: Theoretical directions and an agenda for research. Inf. Syst. J..

[10] Mazmanian M., Orlikowski W.J., Yates J. (2013). The Autonomy Paradox: The Implications of Mobile Email Devices for Knowledge Professionals. Organ. Sci..

[11] Bondanini G., Giorgi G., Ariza-Montes A., Vega-Munoz A., Andreucci-Annunziata P. (2020). Technostress Dark Side of Technology in the Workplace: A Scientometric Analysis. Int. J. Environ. Res. Public Health.

[12] La Torre G., Esposito A., Sciarra I., Chiappetta M. (2019). Definition, symptoms and risk of techno-stress: A systematic review. Int. Arch. Occup. Environ. Health.

[13] Fischer T., Riedl R. (2017). Technostress Research: A Nurturing Ground for Measurement Pluralism?. Commun. Assoc. Inf. Syst..

[14] Moher D., Liberati A., Tetzlaff J., Altman D.G., Group P. (2009). Preferred reporting items for systematic reviews and meta-analyses: The PRISMA statement. PLoS Med..

[15] Borle P., Reichel K., Voelter-Mahlknecht S. (2021). Is There a Sampling Bias in Research on Work-Related Technostress? A Systematic Review of Occupational Exposure to Technostress and the Role of Socioeconomic Position. Int. J. Environ. Res. Public Health.

[16] Mattioli S., Zanardi F., Baldasseroni A., Schaafsma F., Cooke R.M., Mancini G., Fierro M., Santangelo C., Farioli A., Fucksia S. (2010). Search strings for the study of putative occupational determinants of disease. Occup. Environ. Med..

[17] Ouzzani M., Hammady H., Fedorowicz Z., Elmagarmid A. (2016). Rayyan-a web and mobile app for systematic reviews. Syst. Rev..

[18] Nimrod G. (2018). Technostress: Measuring a new threat to well-being in later life. Aging Ment. Health.

[19] Ipsen C., Karanika-Murray M., Nardelli G. (2020). Addressing mental health and organisational performance in tandem: A challenge and an opportunity for bringing together what belongs together. Work Stress.

[20] Berg-Beckhoff G., Nielsen G., Ladekjær Larsen E. (2017). Use of information communication technology and stress, burnout, and mental health in older, middle-aged, and younger workers–results from a systematic review. Int. J. Occup. Environ. Health.

[21] Bramer W.M., Giustini D., de Jonge G.B., Holland L., Bekhuis T. (2016). De-duplication of database search results for systematic reviews in EndNote. J. Med. Libr. Assoc..

[22] Herzog R., Alvarez-Pasquin M.J., Diaz C., Del Barrio J.L., Estrada J.M., Gil A. (2013). Are healthcare workers’ intentions to vaccinate related to their knowledge, beliefs and attitudes? A systematic review. BMC Public Health.

[23] Stadin M., Nordin M., Brostrom A., Hanson L.L.M., Westerlund H., Fransson E. (2019). Repeated exposure to high ICT demands at work, and development of suboptimal self-rated health: Findings from a 4-year follow-up of the SLOSH study. Int. Arch. Occup. Environ. Health.

[24] Stadin M., Nordin M., Broström A., Magnusson Hanson L.L., Westerlund H., Fransson E.I. (2016). Information and communication technology demands at work: The association with job strain, effort-reward imbalance and self-rated health in different socio-economic strata. Int. Arch. Occup. Environ. Health.

[25] Goetz T.M., Boehm S.A. (2020). Am I outdated? The role of strengths use support and friendship opportunities for coping with technological insecurity. Comput. Hum. Behav..

[26] Suh A., Lee J. (2017). Understanding teleworkers’ technostress and its influence on job satisfaction. Internet Res..

[27] Florkowski G.W. (2019). HR technologies and HR-staff technostress: An unavoidable or combatable effect?. Empl. Relat..

[28] Day A., Paquet S., Scott N., Hambley L. (2012). Perceived Information and Communication Technology (ICT) Demands on Employee Outcomes: The Moderating Effect of Organizational ICT Support. J. Occup. Health Psychol..

[29] Lee A.R., Son S.M., Kim K.K. (2016). Information and communication technology overload and social networking service fatigue: A stress perspective. Comput. Hum. Behav..

[30] Wu J., Wang N., Mei W., Liu L. (2020). Technology-induced job anxiety during non-work time: Examining conditional effect of techno-invasion on job anxiety. Int. J. Netw. Virtual Organ..

[31] Jena R.K. (2015). Technostress in ICT enabled collaborative learning environment: An empirical study among Indian academician. Comput. Hum. Behav..

[32] Khedhaouria A., Cucchi A. (2019). Technostress creators, personality traits, and job burnout: A fuzzy-set configurational analysis. J. Bus. Res..

[33] Kim H.J., Lee C.C., Yun H., Im K.S. (2015). An examination of work exhaustion in the mobile enterprise environment. Technol. Forecast. Soc. Chang..

[34] Gaudioso F., Turel O., Galimberti C. (2017). The mediating roles of strain facets and coping strategies in translating techno-stressors into adverse job outcomes. Comput. Hum. Behav..

[35] Lee J. (2016). Does stress from cell phone use increase negative emotions at work?. Soc. Behav. Personal..

[36] Al-Ansari M.A., Alshare K. (2019). The Impact of Technostress Components on the Employees Satisfaction and Perceived Performance: The Case of Qatar. J. Glob. Inf. Manag..

[37] Tarafdar M., Pullins E.B., Ragu-Nathan T.S. (2015). Technostress: Negative effect on performance and possible mitigations. Inf. Syst. J..

[38] Tarafdar M., Tu Q., Ragu-Nathan T.S. (2011). Impact of technostress on end-user satisfaction and performance. J. Manag. Inf. Syst..

[39] Alam M.A. (2016). Techno-stress and productivity: Survey evidence from the aviation industry. J. Air Transp. Manag..

[40] Vayre E., Vonthron A.M. (2019). Identifying Work-Related Internet’s Uses—at Work and Outside Usual Workplaces and Hours—and Their Relationships With Work–Home Interface, Work Engagement, and Problematic Internet Behavior. Front. Psychol..

[41] Molino M., Cortese C.G., Ghislieri C. (2020). The Promotion of Technology Acceptance and Work Engagement in Industry 4.0: From Personal Resources to Information and Training. Int. J. Environ. Res. Public Health.

[42] Baruch Y. (2000). Teleworking: Benefits and pitfalls as perceived by professionals and managers. New Technol. Work Employ..

[43] Orlikowski W., Baroudi J. (1991). Studying Information Technology in Organizations: Research Approaches and Assumptions. Inf. Syst. Res..

[44] Ma J., Ollier-Malaterre A., Lu C.-q. (2021). The impact of techno-stressors on work–life balance: The moderation of job self-efficacy and the mediation of emotional exhaustion. Comput. Hum. Behav..

[45] Yener S., Arslan A., Kilinç S. (2020). The moderating roles of technological self-efficacy and time management in the technostress and employee performance relationship through burnout. Inf. Technol. People.

[46] Pirkkalainen H., Salo M., Tarafdar M., Makkonen M. (2019). Deliberate or Instinctive? Proactive and Reactive Coping for Technostress. J. Manag. Inf. Syst..

[47] Parker S.K., Grote G. (2020). Automation, Algorithms, and Beyond: Why Work Design Matters More Than Ever in a Digital World. Appl. Psychol..

[48] Borle P., Boerner-Zobel F., Voelter-Mahlknecht S., Hasselhorn H.M., Ebener M. (2020). The social and health implications of digital work intensification. Associations between exposure to information and communication technologies, health and work ability in different socio-economic strata. Int. Arch. Occup. Environ. Health.

[49] Sterling T.D. (1959). Publication Decisions and Their Possible Effects on Inferences Drawn from Tests of Significance—Or Vice Versa. J. Am. Stat. Assoc..

[50] Rosenthal R. (1979). The “File Drawer Problem” and Tolerance for Null Results. Psychol. Bull..

[51] Tams S., Hill K. (2017). Helping an old workforce interact with modern IT: A NeuroIS approach to understanding technostress and technology use in older workers. Lect. Notes Inf. Syst. Organ..

[52] Riedl R. (2013). On the Biology of Technostress: Literature Review and Research Agenda. Data Base Adv. Inf. Syst..

